# Rapid Spontaneous Resolution of Fibromatosis Colli in a 3-Week-Old Girl

**DOI:** 10.1155/2014/264940

**Published:** 2014-01-12

**Authors:** Paolo Adamoli, Piero Pavone, Raffaele Falsaperla, Roberta Longo, Giovanna Vitaliti, Claudio Andaloro, Serra Agostino, Salvatore Cocuzza

**Affiliations:** ^1^Unit of Pediatrics, “Moriggia-Pelascini” Hospital, Via Pelascini, 3, 22015 Gravedona ed Uniti Como, Italy; ^2^Department of Pediatrics and Pediatric Emergency “Costanza Gravina”, University Hospital “Vittorio Emanuele-Policlinico”, Via Plebiscito 7674, 95124 Catania, Italy; ^3^Department of Medical Surgical Specialties, ENT Clinic, University Hospital “Vittorio Emanuele-Policlinico”, Via Santa Sofia, 78, 95123 Catania, Italy

## Abstract

Fibromatosis colli is an uncommon benign, congenital fibrous tumor or pseudotumor of the sternocleidomastoid muscle that manifests in infancy. In some of these patients tightening of the muscle results in torticollis. We report the case of a 3-week-old child, who presented with a neck mass localized in the left side with reduced mobility of the head. The diagnosis of fibromatosis colli was raised by ultrasound sonography. The mass regressed spontaneously within 3 months without surgical or physical treatment.

## 1. Introduction

Fibromatosis colli, also known as “sternocleidomastoid pseudotumor of infancy,” is an uncommon benign lesion of the spindle cells of the sternocleidomastoid (SCM) muscle [1–6]. The term “tumour” is a misnomer because it is not a cancerous condition but it is referred to as a congenital fibrotic process, so in this particular context the word tumour simply means swelling. This lesion affects infants with an incidence of 0.4%. It is usually unilateral, affects the right side in 75% of cases, and affects male patients slightly more often than female patients [5, 6]. History of complicated delivery and birth injury is associated in more than 50% of cases [2–6]. It typically presents as a palpable solid mass of the anterior part of the neck over the SCM muscle. The mass is not present at birth but tends to appear between the 2nd and 4th week of life. We present here the case of a 3-week-old female child who showed a swelling left laterocervical tumefaction and hypomobility of the head from the first weeks of life. The ultrasound (US) of the lesion led to the diagnosis of fibromatosis colli. A new ultrasound examination performed at the age of 3 months showed a rapid regression of the lesion without the need of any kind of treatment.

## 2. Case Report

**Figure 1:**
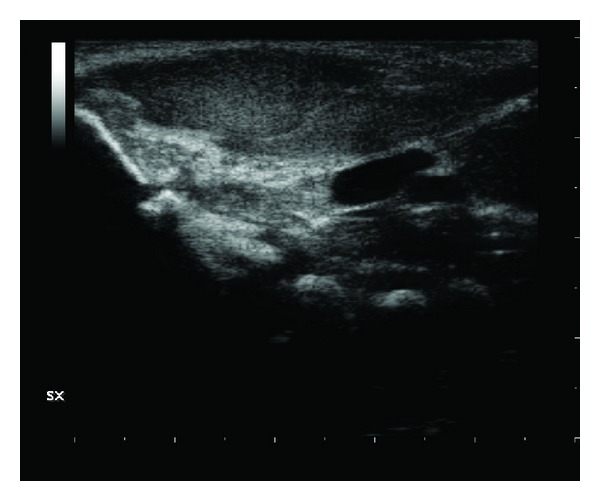
At the left side in the laterocervical region, there is a fusiform thickening of the ventral side of the the sternocleidomastoid muscle.

**Figure 2:**
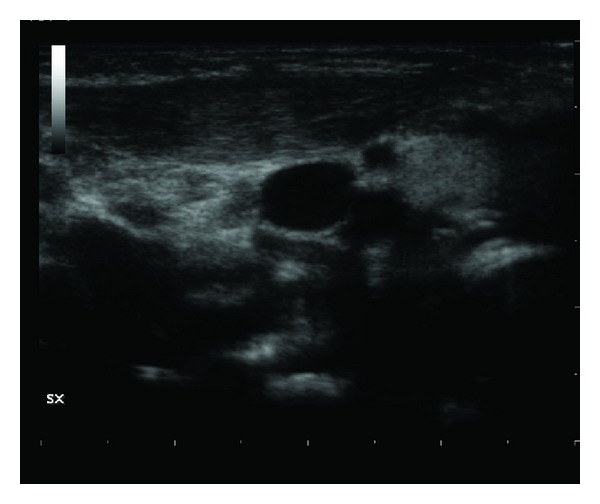
The ultrasound scan made after a month shows a marked improvement of the sternocleidomastoid muscle, with only an increase of myofiber in the third middle of the ventral belly.

A 3-week-old girl was referred by her pediatrician to the “Pediatric Operative Unit” of the Hospital “Moriggia-Pelascini,” Gravedona, Como, Italy, for an ultrasound evaluation of a tumefaction of the neck. The girl was born after forceps delivery at 38 weeks of gestation, with a birth-weight of 2,950 gr, height of 49 cm, and head circumference of 35 cm. Family history was negative for fibrous tissue tumors or congenital torticollis. The girl is the first born child of healthy nonconsanguineous parents. The mother denied having had infectious diseases during pregnancy and having taken drugs or alcohol. For personal reasons the family moved to Catania and the girl was admitted to the Pediatric Operative Unit of Hospital Vittorio Emanuele, Catania, Italy. On physical examination, the girl showed weight, length, and head circumference within normal limits, and no malformations were noticed. There was a nontender neck swelling on the left side located in the lower and middle third of sternal portion of the SCM muscle, soft in consistency and not warm to touch with reduced neck movement on the affected side. In order to determine the severity of limitation of passive neck rotation, gentle neck rotation to the left was performed with the baby supine and head held over the side of the examining table, and a limited rotation was noted which can be graded as mild − moderate *≈*70 degrees (normal 90–110 degrees) from the neutral position in opposition to the normal right neck rotation *≈*100 degrees. No cervical lymphadenopathy was present. The girl was otherwise normal, feeding at the breast, and the body temperature was normal. The routine laboratory tests were normal. The US examination documented a thyroid gland of normal size and echostructure. The left lobe of the gland was displaced superiorly from the mediastinum by the protrusion of the thymic tissue. The parotid and salivary glands were normal. At the left side in the laterocervical region, US showed a fusiform homogeneous thickening of the ventral side of the SCM muscle (Figure 1). The fusiform shaped mass, which moved synchronously with the muscle, appeared isoechoic to the rest of the muscle. In comparison, the right sternocleidomastoid muscle appeared normal. No significant change in internal vascularity was seen. The fibrillar structure of the muscle fibers was however maintained. There was no cervical lymphadenopathy and no vascular invasion or bony involvement as may be seen with other neck masses. Based on the clinical and US findings, a diagnosis of fibromatosis colli was made. The girl was regularly followed up every month. At the age of 3 months the tumefaction progressively regressed and the movements of the neck became regular. US made at this age showed a remarkable improvement of the lesion with only an increase of myofiber in the third needle of the ventral belly (Figure 2), with an almost complete disappearance of the tumefaction.

## 3. Discussion

The infant, born after forceps delivery, in the second week of life presented with a tumefaction localized in the left laterocervical area of the neck. Lateral movement of the head was reduced. No other clinical anomalies were noticed. The US showed a lesion typical of fibromatosis colli or pseudo-tumor of the sternocleidomastoid muscle. Fibromatosis colli was first described as “sternomastoid tumor torticollis” in German literature in 1812 by Hulbert [3]. It was characterized by Chandler and Altenberg [4] as the appearance of “a hard, immobile, fusiform swelling in the sternomastoid muscle which usually is detected at 2 weeks after birth and then increases in size for two to four weeks until it reaches the size of a very large almond.” The mean ages of presentation are 24 days. They are usually common unilaterally but rarely found bilaterally. Fibromatosis colli is more common on the right (73%–75%) than the left side of the neck (22%) [5, 6]. However, our patient had the lesion on the left side of the neck. In typical fibromatosis colli, a mass is present at 1–8 weeks of age with torticollis and a non-tender, firm fusiform swelling within the body of the SCM muscle. The mass may increase in size for several weeks (growth phase), then stabilize in size for a few months, and finally diminish spontaneously by 4–8 months of age [7]. The lesion incorporated in the SCM muscle, usually in the distal one-third, is mobile and palpable beneath the skin. It is a benign condition which in about 80% of cases gradually decreases and completely resolves. Sometimes the condition may cause torticollis which is reported in 14%–20% of cases [8, 9]. Although the pathogenesis of this lesion is not clear, it is probably related to the trauma of birth for prolonged difficult labour; breech presentation, forceps delivery, and primiparous birth have been implicated. One of the most plausible hypotheses is an obstructed venous outflow in the muscle either during delivery or during intrauterine development [10–12]. The injury leads to necrosis and then fibrosis in the muscle fibers resulting in the development of secondary pressure within the muscle. Davids et al. [8] have suggested that the lesion results from an in utero fetal head position, which causes selective injury to the SCM muscle. Such an injury leads to the development of a secondary compartment syndrome and resultant pressure necrosis and fibrosis within the muscle [10–12].

Lateral cervical swelling during early childhood and especially during the first month of life is a clinical sign typically suggestive of fibromatosis colli. Neck reactive lymph nodes, abscesses, bronchogene cysts and thyroid lobe dislocation are clinically easily distinguished from fibromatosis as well as malignant conditions such as neuroblastoma, lymphoma, rhabdomyosarcoma, teratoma and fibrosarcoma [10] and benign neoplasmatic conditions such as hemangioma and cystic hydroma. In the differential diagnosis also vascular malformation (particularly lymphatic malformation/lymphangioma), beside bronchogenic cysts and thyroglossal duct cysts, is to be evaluated. Abscess is less likely in a small baby but more likely in the setting of immunedeficiency or in older children. Although the fibromatosi colli is a disease that can lead to respiratory failure and subsequently a tracheostomy, such evidence has never yet been reported in the literature [11].

Most authors report, in agreement with our findings, that US is the preferred diagnostic tool because of its easy availability, low cost, and lack of ionizing radiation [12]. The SCM muscle is diffusely enlarged in a fusiform manner, with resultant shortening and therefore head turned away from the affected side (mastoid process drawn down towards the ipsilateral head of clavicle). Echogenicity may vary. Colour Doppler interrogation may reveal a high resistance waveform. The enlarged area often moves synchronously with the rest of the SCM muscle on real-time sonography [13]. Though US is the imaging modality of choice, cross-sectional imaging with CT scan or MRI may occasionally be used to exclude other conditions when the clinical findings are equivocal or atypical and may be required to further characterize the disease and to find out the extent of involvement. CT scans typically shows a diffusely enlarged SCM muscle which is isoattenuating to normal neighbouring musculature. Adjacent fat planes are well preserved. At times calcification may be present [14]. MRI shows a well-defined mass with increased signal on T1 weighted and T2 sequences surrounded by tissue with normal muscle signal. Treatment of fibromatosis colli is conservative; rarely physiotherapy or surgical intervention by tenomyotomy or tenotomy is required [15, 16].

## 4. Conclusion

The scientific literature reports a spontaneous resolution of the lesion over a period of 4 to 8 months.

In our case the lesion disappeared in a few months (3 months) without any kind of treatment. We explain that because in very young child, as our case, the degeneration of the muscle fiber and replacement with fibrosis tissue are not with collagen scarlike tissue, typical of the older children, but with immature fibrous tissue that may regenerate more quickly such as normal muscle fibers compared with collagen scar-liketissue. Moreover in this case report the extent of the fibrotic change of the muscle showed by US does not affect the entire length of the SCM muscle, but it is limited to the ventral side; this may lead eventually to a spontaneous resolution more easily and quickly. We agree that in some cases physiotherapy may be useful to avoid the torticollis complication. However, we advise US monitoring of affected patients and checking the gradual decrease of the tumefaction; in this case any kind of treatment can be avoided.
